# Impact of vitamin E and selenium supplementation on growth, reproductive performance, and oxidative stress in dexamethasone-stressed Japanese quail cocks: Vitamin E & selenium in stressed quail cocks

**DOI:** 10.1016/j.psj.2025.104888

**Published:** 2025-02-05

**Authors:** Ifeanyi Emmanuel Uzochukwu, Luke Chukwudi Ali, Bright Chigozie Amaefule, Chisom C. Okeke, Charles Onochie Osita, Ndubuisi Samuel Machebe, Vesela Yancheva, Dóra Somogyi, Krisztián Nyeste

**Affiliations:** a Department of Animal Science, University of Nigeria, Nsukka, Enugu, Nigeria; b Department of Hydrobiology, University of Debrecen, P.O. Box 57, Debrecen 4010, Hungary; c Pál Juhász-Nagy Doctoral School of Biology and Environmental Sciences, University of Debrecen, Debrecen, Hungary; d Department of Ecology and Environmental Conservation, Faculty of Biology, Plovdiv University, Plovdiv 4000, Bulgaria; e National Laboratory for Water Science and Water Security, University of Debrecen, Debrecen, Hungary

**Keywords:** Dexamethasone-induced stress, Oxidative stress response, Antioxidants, Micronutrient supplementation, Japanese quail reproduction

## Abstract

This study investigated the effects of dietary vitamin E (VE) and selenium (Se) supplementation on body weight changes, blood profile, and semen quality in Dexamethasone (DEX)-stressed Japanese quails. One hundred and five 10-week-old quail cocks were acclimated and divided into five treatment groups: negative control – G1, DEX-treated (20 mgL^−1^ of drinking water) – G2, DEX + VE (180 mg kg diet^−1^) – G3; DEX + Se (0.3 mg kg diet^−1^) – G4; and DEX + VE (180 mg kg diet^−1^) + Se (0.3 mg kg diet^−1^) – G5. The birds received their respective treatments over 21 days, and various performance, hematological, and semen quality parameters were measured. Results indicated that DEX treatment significantly reduced weight gain (WG) and feed intake (*P* < 0.05). Supplementation with VE and Se, individually and combined, ameliorated these effects, with groups G3, G4, and G5 showing similar WG to the control. Hematological analysis revealed significant increases (*P* < 0.05) in packed cell volume, hemoglobin, and white blood cell count in DEX-treated groups compared to G1. Treatment did not affect blood glucose and cholesterol levels (*P* ≥ 0.05). Plasma antioxidant assays showed elevated superoxide dismutase and catalase functions and reduced malondialdehyde levels in G3, G4, and G5 compared to G2, indicating reduced oxidative stress. No marked differences were seen in the plasma glutathione peroxidase activities across groups. Sperm motility was impaired in the DEX-only group but improved (*P* < 0.05) with antioxidant supplementation. In conclusion, dietary VE and Se effectively mitigated the negative impacts of DEX-induced stress on growth, antioxidant status, and spermatozoa motility in Japanese quail cocks. VE and Se supplementation could be beneficial in enhancing the welfare and productivity of poultry under stress.

## Introduction

Japanese quail (*Coturnix coturnix japonica* Temminck and Schlegel, 1848) rearing, or coturniculture, has grown in popularity given its high productivity and profitability. These birds are known for their rapid growth cycles (3-4 generations per year), disease resistance, and manageable size, making them ideal for meat production and research models (Seker et al., 2009; Saki et al., 2012; Ratriyanto et al., 2020). However, heat stress (**HS**) poses a significant challenge, particularly in tropical regions. High temperatures and humidity disrupt quail health and productivity by increasing lipid peroxidation, energy demands, and compromising cell membranes (Tumová and Gous, 2012; Hosseini-Vashan and Raei-Moghadam, 2019; Masuda et al., 2024). Previous studies documented elevated oxidative stress markers and reduced antioxidant levels in Japanese quails exposed to HS (32-34°C) (Costantini et al., 2014; Orhan et al., 2020; Truong and King, 2023). Climate change further exacerbates these issues, highlighting the need for effective HS mitigation strategies.

Oxidative stress (**OS**), referring to an imbalance between free radicals and antioxidants, contributes to stress-induced reproductive and productivity declines in quails and chickens (Peters et al., 2020). During stress, birds experience elevated free radicals, leading to OS. Stressful conditions trigger the activation of the hypothalamic-pituitary-adrenal axis, resulting in an increased glucorticoid (avian corticosterone) secretion to maintain homeostasis (Chung et al., 2011). However, excess glucocorticoids can negatively impact growth and immunity and induce OS (Coutinho and Chapman, 2011). Dexamethasone (**DEX**), a synthetic glucocorticoid, mimics elevated corticosterone levels and triggers stress pathways in avian research (Shini et al., 2010; Calefi et al., 2016; Pan et al., 2019; Osho and Adeola, 2020; Dai et al., 2022).

Dietary supplementations with antioxidants like vitamin E (**VE**) and selenium (**Se**) offer a promising strategy to combat OS and its detrimental effects on poultry species (El-Senousey et al., 2018). VE as a natural antioxidant, scavenges free radicals, protecting tissues and playing crucial roles in metabolism (Jang et al., 2014; Amevor et al., 2021). It enhances antioxidant capacity and immune function (Khan et al., 2011) and mitigates the negative impacts of DEX exposure on avian semen (Eid et al., 2006). VE supplementation can also improve poultry performance and reduce OS under elevated temperature settings (Attia et al., 2017).

Se is an important micronutrient that supports numerous biological activities like antioxidation, anti-inflammation, and immune system modulation (Liu et al., 2017; Mechora et al., 2017; Ventura et al., 2017; Qazi et al., 2019). It functions as a component and cofactor of antioxidant enzymes like Glutathione peroxidase (**GSH-Px**), superoxide dismutase (**SOD**), and catalase (**CAT**), protecting cells from free radical damage (El-kazaz et al., 2020). Se supplementation can enhance testicular Se levels, boosting seminal antioxidant activity and reducing lipid peroxidation, ultimately enhancing semen quality (El-kazaz et al., 2020). Furthermore, VE and Se work synergistically to improve antioxidant capacity, general health, and semen quality, including increased sperm viability and reduced morphological abnormalities (Edens and Sefton, 2009; Celebi, 2019).

The administration of DEX has been widely used to simulate the physiological effects of glucocorticoids and stress in quails and other avian species, with numerous studies documenting its impacts (Hanafy and Hassan, 2021; Crouch et al., 2022). Additionally, extensive research has explored the antioxidant benefits of VE and Se, both individually and in combination with other nutrients, in mitigating OS in physiologically or heat-stressed Japanese quail (El-Kazaz et al., 2020; Gouda et al., 2020; Astuti et al., 2024), chickens (Gao et al., 2010; Jang et al., 2014; El-Senousey et al., 2018; Khalil-Khalili et al., 2020), and even dogs (Hatamoto et al., 2006). However, the physiological effects of combined VE and Se supplementation in Japanese quail exposed to chronic physiological stress remain underexplored.

Most studies have primarily focused on the impact of these antioxidants on growth and physiological responses in DEX-treated or heat-stressed chickens (Harsini et al., 2012; Habibian et al., 2014; Ademu et al., 2024; Bora et al., 2024). While some research has examined the effects of VE and Se supplementation in heat-stressed quails (Sahin and Kucuk, 2001; Sahin et al., 2002), studies specifically investigating the impact of these antioxidants in chronic-stressed or DEX-treated quails remain limited. Therefore, this study aims to investigate the combined effects of VE and Se supplementation on growth, hematological parameters, plasma antioxidant status, seminal characteristics, and oxidative stress in DEX-stressed quail cocks. By addressing this gap in the literature, our research seeks to provide valuable insights into the potential synergistic benefits of these antioxidants in enhancing the health and reproductive performance of Japanese quail under chronic stress conditions. As a valuable model for studying avian biology and reproductive performance, Japanese quail presents a unique model for understanding the interplay between stress and nutritional interventions. This understanding can inform the development of effective nutritional strategies to enhance the welfare and productivity of poultry under stress conditions, particularly in tropical regions.

## Materials and methods

### Research ethics

All animal procedures adhered to ethical guidelines provisions of the University of Nigeria, Nsukka Research Policy Document (Research Ethics Committee Recommendations, 2013). Every effort was made to minimize animal discomfort.

### Experimental site and microclimate

**Fig. 1:**
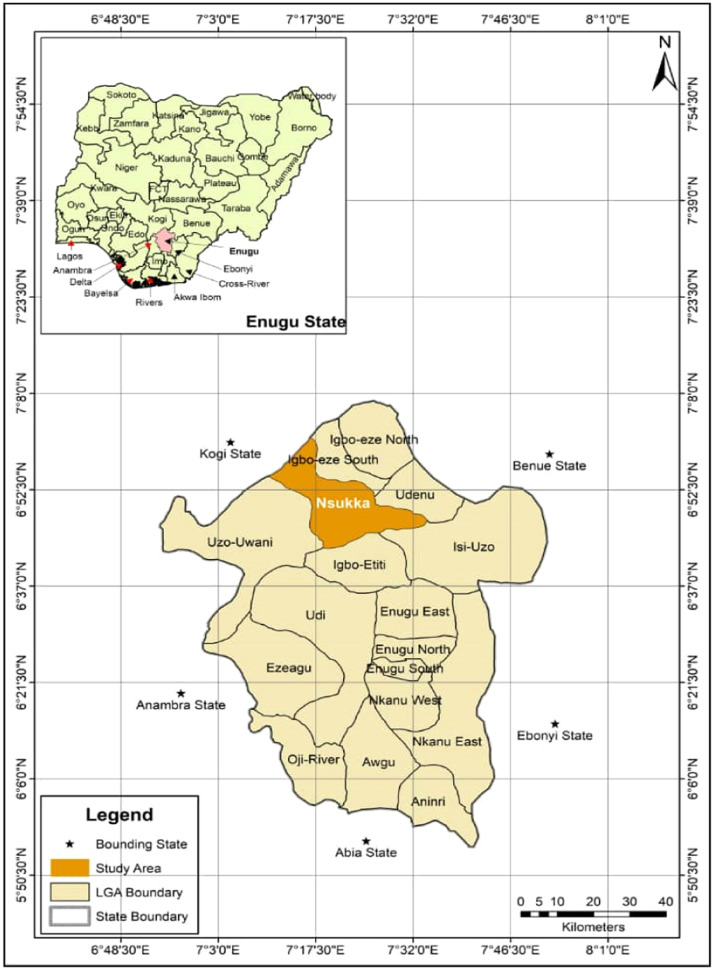
Map of the University of Nigeria Farm showing the experimental site.

The experiment was conducted in natural tropical environmental settings at the poultry section of the Department of Animal Science Teaching and Research Farm, University of Nigeria, located in Nsukka Local Government Area, Enugu State (latitude 6° 51′39″ N and longitude 7° 21′39″ E) (Fig. 1). The experimental location features a humid tropical microclimate with a bimodal annual rainfall ranging from 1155 to 1955 mm, as Uguru et al. (2011) reported. The average ambient temperature observed during the study was 30.58°C, with a mean relative humidity of 71.7 %. The study area's photoperiodicity was a natural 12 h light and 12 h dark cycle, with no extra lighting system applied.

### Choice of DEX administration method

Dexamethasone (DEX), a synthetic glucocorticoid, mimics elevated corticosterone levels and activates stress pathways in avian species (Shini et al., 2010; Calefi et al., 2016; Pan et al., 2019; Osho and Adeola, 2020; Dai et al., 2022). External adrenocorticotropic hormone (ACTH) administration is commonly used to study the dynamics of hypothalamic-pituitary-adrenal (HPA) axis hormones (Wilson and Holberton, 2001). In larger species, invasive methods like intramuscular or intrajugular delivery are standard. However, in smaller species with limited muscle mass, less invasive techniques, such as administration via feed, drinking water, or intraperitoneal injection, are recommended (Wilson and Holberton, 2001).

Previous studies using intraperitoneal (Wilson and Holberton, 2001), intramuscular (Crouch et al., 2022), or dietary DEX administration (Hanafy and Khalil, 2015; Berenjian et al., 2018; Hanafy and Hassan, 2021; Yarmohammadi et al., 2020) in smaller birds focused primarily on short-term or acute stress responses. To investigate the physiological impact of chronic DEX exposure, this study was modelled on Lv et al. (2018), who reported that administering 20 mg/L DEX to broiler chicks significantly reduced feed intake and body weight gain, altered liver malondialdehyde (MDA) levels, superoxide dismutase (SOD) activity, and the mRNA expression of CuZn-SOD, Mn-SOD, and glutathione peroxidase (GSH-Px). Chronic exposure (days 19–41) also disrupted serum metabolites and increased mortality.

Given that Japanese quail are more resilient to stress, particularly heat stress, compared to chickens (Barbosa Lima et al., 2014; Alagawany et al., 2017), we adopted and adapted this protocol for adult quail. The study included both negative and positive control groups to comprehensively assess the physiological effects of chronic DEX administration on Japanese quail cocks.

### Management and treatment of experimental cocks

One hundred and five 10-week-old male Japanese quails (mean body weight = 204.55 ± 2.11 g) were sourced from the National Veterinary Research Institute, Vom, Jos, Nigeria. Upon arrival, the birds underwent a thorough health inspection. They were administered a commercially available anti-stress vitamin supplement (SuperVit Plus) for two weeks to minimize stress during acclimation. Following acclimation, birds were randomly distributed into five treatment groups (*n* = 7 birds/replicate; 3 replicates/group) housed in individual wooden cages measuring 281 cm (length) x 175 cm (breadth) x 86 cm (height). Quails were fed a commercially formulated quail breeder ration designed to meet the National Research Council (1994) recommendations of 21 % crude protein and 2.90 Mcal/kg metabolizable energy (composition details provided in Table 1). The five treatment groups were as follows:

• Group 1 (G1): Negative control – Basal diet with no supplementation.
• Group 2 (G2): Positive control – Basal diet + **DEX**.
• Group 3 (G3): Basal diet + DEX + 180 mg/kg dietary vitamin E (VE, d-alpha Tocopheryl Acetate) supplementation.
• Group 4 (G4): Basal diet + DEX + 0.3 mg/kg dietary organic selenium (Se) supplementation.
• Group 5 (G5): Basal diet + DEX + combined dietary supplementation of 180 mg/kg VE and 0.3 mg/kg Se.

**Table 1:** Basal compositions of experimental diet.

Ingredients	Compositions (%)
Maize	18
Wheat offal	13
Cassava flour	20
Indomie	15
Soybean meal	12
Groundnut cake	13
Fish meal	5
Lysine	1
Methionine	0.6
Premix*	0.5
Salt	0.4
Calculated Chemical Composition	
Crude Protein %	21
Crude Fibre %	5.5
Metabolizable Energy (Mcal/kg)	2.90

⁎ Each 1 kg of premix contains: 10,000 IU vitamin (vit.) A; 62.5 μg cholecalciferol; 44.4 IU vit. E; 3 mg vit. K3; 2 mg thiamine; 5 mg riboflavin; 5 mg pyridoxine; 0.015 mg vit. B12; 40 mg Nicotinic acid; 12 mg Pantothenic acid; 0.75 mg folic acid; 0.05 mg biotine; 100 mg vit. C; 70 mg manganese/kg; 60 mg zinc/kg; 60 mg iron/kg; 1 mg iodine/kg; 8 mg copper/kg; 0.25 mg selenium/kg; and 0.15 mg cobalt/kg.

DEX (dexamethasone sodium phosphate) was administered through the water (at a dosage of 20 mg/L) (adapted from Lv et al., 2018) and given to the treated groups every other day. The DEX treatment and feeding trial lasted for 21 days. Birds were continuously provided access to their assigned diets and fresh drinking water throughout the experiment. Furthermore, all experimental groups underwent similar management practices to ensure treatment consistency.

### Sample collection and analysis

***Body weight measurements.*** The individual cock's body weight (**BW**) was measured using a calibrated electronic balance (Camry Digital Weighing Scale; Model: EI-02HS) with an accuracy of 0.01 g. Measurements were taken twice: before the trial's commencement and at the experiment's end. BW gain was calculated by subtracting the average initial BW of birds in each group from their average final BW. Weekly feed intake (**FI**) was determined by calculating the difference between the weight of the feed initially provided to each cage and the weight of the leftover feed, following previously described methods (El-kazaz et al., 2020).

***Blood Collection, Hematology, and Plasma Biochemistry Analysis****.* The birds were fasted before the blood collection exercise. Blood samples were taken after the trial from the cocks’ jugular veins into test tubes containing EDTA (1 mg/ml) for hematological analysis (2 ml) and into non-heparinized tubes for plasma biochemical tests (2 ml). The heparinized tubes were capped with rubber stoppers and gently rocked to ensure uniform mixing with the anticoagulant. Hematological indices, including hemoglobin (**Hb**), packed cell volume (**PCV**), red blood cell (**RBC**) count, white blood cell (**WBC**) count, and WBC differentials (heterophils, lymphocytes, monocytes, eosinophils, and basophils), were determined according to the methods described by Coles (1986).

Following a 10-minute centrifugation at 3000 rpm, the obtained plasma supernatant was carefully transferred and preserved at −20°C for later analysis. Blood glucose concentrations were obtained using an Accu-Chek Guide Meter (Roche Diagnostics, Indianapolis, IN, USA) according to the manufacturer's protocol. Plasma cholesterol was measured by Technicon RA-XT biochemical analyzer (Technicon Corporation, Tarrytown, NY). The plasma levels of SOD, CAT, GSH-Px, and Malondialdehyde (**MDA**) were assessed spectrophotometrically using specific commercial biodiagnostic ELISA kits and following their manufacturer's (Elabscience Biotechnology Co., Ltd., Houston, TX, USA) instructions.

***Semen Evaluation and Seminal Oxidative Assay.*** At the conclusion of the study, semen samples were collected from all treatment groups for evaluation. Before semen collection, the cocks were trained and familiarized with human handling. Semen collection was carried out using the dorso-abdominal massage technique by two individuals, as described by Castillo et al. (2021). Briefly, individual cocks were first restrained, and the cloacal foam was removed. Dorsoabdominal massages (3-4x) were applied to the lumbar region while maintaining vent pressure. Ejaculated semen was aspirated with a calibrated micropipette following pre-collection cleaning of the vent area with sterile tissue to minimize contamination. The semen volume was directly read off on the micropipettes. The semen was then diluted with normal saline (NaCl 0.9 %, w/v) and evaluated for sperm motility using a microscope at x400. The determination of the physical semen characteristics, including motility, viability, dead sperm cells, and sperm concentrations was made as described by Uzochukwu et al. (2019). Sperm concentrations were measured using a Neubauer hemocytometer (Rodriguez-Martinez, 2013) and expressed as spermatozoa × 10^6^/ml. To assess sperm viability and mortality, a drop of semen was placed on a glass slide, combined with a drop of eosin-nigrosin stain, spread using another slide, air-dried, and observed under a microscope at 400x magnification.

For the seminal oxidative assay, diluted semen samples from the respective treatment groups were centrifuged at 3000 rpm for 10 min to obtain the seminal plasma. The seminal plasma was assayed for GSH-Px and MDA using commercial kits and procedures similar to those utilized for the blood plasma assay. The selection of sGSH-Px and sMDA as indices of semen quality in this study arises from their functions in antioxidant defence and as biomarkers of oxidative stress in sperm physiology. In particular, sGSH-Px plays a vital role in alleviating oxidative stress damage in sperm cells (Otasevic et al., 2019), while sMDA serves as a significant indicator of lipid peroxidation, with elevated levels correlated with reduced semen quality in males (Collodel et al., 2015).

### Data analysis

The map (Fig. 1) was created using QGIS Software (QGIS Geographic Information System, 2024). Before analysis, the data were evaluated for normality and variance homogeneity using the Shapiro-Wilk and Levene's tests, respectively. A one-way analysis of variance (ANOVA) was carried out to assess differences among groups, with Duncan's multiple range test used for pairwise comparisons where applicable. All statistical analyses were performed with IBM SPSS Statistics version 20 (IBM Corp., 2011), with significance established at *p* < 0.05. The plasma antioxidant enzymes and MDA charts were made using the ggplot2 package in RStudio (RStudio Team, 2019).

## Results

### Body weight and feed intake

The results of body weight and FI measurements (± Standard Error of Mean, **SEM**) of DEX-stressed quail cocks fed dietary VE and Se supplementation are shown in Table 2. There were no significant changes in initial body weight (**IBW**) (*P* = 1.00) and final body weight (**FBW**) (*P* = 0.678) across the treatment groups. However, weight gain (WG), total feed intake (TFI), and daily feed intake (**DFI**) were markedly influenced by the dietary treatments (*P* < 0.05).

**Table 2:** Effect of vitamin E and selenium supplementation on body weight changes (SEM) of DEXamethasone stressed quail cocks.

Indices	Group 1 (Control)	Group 2 (DEX)	Group 3 (DEX + VE)	Group 4 (DEX + Se)	Group 5 (DEX + VE + Se)	P-value
IBW (g)	218.48 ± 3.90	219.41 ± 3.23	219.30 ± 4.75	219.14 ± 3.31	218.80 ± 3.22	1.000^NS^
FBW (g)	234.85 ± 1.59	228.49 ± 4.09	230.78 ± 3.59	233.12 ± 3.24	231.88 ± 2.48	0.678^NS^
WG (g)	16.36 ± 2.34^a^	9.08 ± 0.98^c^	11.48 ± 1.51^b^	13.98 ± 0.55^ab^	13.08 ± 0.77^ab^	0.038*
TFI (g)	405.65 ± 62.86^a^	197.68 ± 17.96^b^	354.90 ± 22.80^ab^	301.00 ± 8.87^ab^	355.74 ± 38.94^ab^	0.019*
DFI (g)	19.32 ± 2.99^a^	9.41 ± 0.86 ^b^	16.90 ± 1.09^ab^	14.33 ± 0.42^ab^	16.90 ± 1.85^ab^	0.019*

^a–c^Row means with distinct alphabets differ significantly at *P* < 0.05 (One-Way ANOVA, Duncan's multiple range test); IBW: Initial body weight, FBW: final body weight, WG: weight gain, DFI: daily feed intake, NS: Not significant, DEX: Dexamethasone; VE: Vitamin E; Se: Selenium.

Cocks in the negative control group (G1) displayed the highest WG and was higher than the DEX-treated group (G2), which exhibited the lowest WG. Notably, WG in Group 3 (G3, DEX + VE) was statistically similar (*P* ≥ 0.05) to that in Groups 4 (G4, DEX + SE) and 5 (G5, DEX + VE + SE), both of which were also similar to G1. Regarding TFI, birds in G1 consumed significantly more feed (*P* < 0.05) than other groups. Conversely, G2 exhibited the lowest TFI (*P* < 0.05). DFI for groups G3, G4, and G5 did not differ (*P* ≥ 0.05) from each other, nor the control or DEX (G2) groups.

### Hematological indices

The hematological indices of PCV, HB, and WBC count varied (*P* < 0.05) across the groups, as shown in Table 3. Other Indices, including RBC and WBC differentials of heterophils, lymphocytes, monocytes, eosinophil, and basophil, and the heterophil: lymphocyte ratio, however, were not markedly affected (*P* ≥ 0.05). Cocks in the negative control group (G1) exhibited the lowest PCV compared to those in G4 and G5, which showed statistically increased PCV (*P* < 0.05). Interestingly, G4 and G5 did not differ from each other (*P* ≥ 0.05), suggesting that both VE alone (G4) and the combination with Se (G5) may contribute to improved PCV. Conversely, the PCV levels in G2 and G3 did not differ significantly (*P* ≥ 0.05) from each other or the other groups. A similar pattern was observed for Hb and WBC. In comparison to the G1, birds in G2, G3, G4, and G5 all displayed significantly higher Hb and WBC levels (*P* < 0.05). Overall, the results show marked increases in the Hb, PCV, and WBC of the DEX-stressed quail cocks.

**Table 3:** Effect of vitamin E and selenium supplementation on hematology (SEM) of dexamethasone stressed quail cocks.

Indices	Group 1 (Control)	Group 2 (DEX)	Group 3 (DEX + VE)	Group 4 (DEX + Se)	Group 5 (DEX + VE + Se)	P-value
PCV (%)	38.25 ± 0.85^b^	41.25 ± 0.48^ab^	40.5 ± 1.19^ab^	43.75 ± 1.31^a^	43.25 ± 1.11^a^	0.012*
HB (g/dl)	10.00 ± 0.52^b^	11.50 ± 0.19^a^	11.67 ± 0.32^a^	12.10 ± 0.21^a^	12.00 ± 0.47^a^	0.007*
RBC (× 10^6^/mm^3^)	9.41 ± 0.28	10.05 ± 0.40	10.66 ± 0.11	10.72 ± 0.43	10.74 ± 0.37	0.054^NS^
WBC (× 10^3^/mm^3^)	8.13 ± 2.50^b^	11.05 ± 3.0^a^	10.70 ± 4.64^a^	10.78 ± 3.64^a^	11.20^a^ ± 3.76	<0.001*
Heterophil (%)	16.00 ± 2.16	20.75 ± 3.25	19.50 ± 1.55	23.00 ± 4.04	19.50 ± 2.10	0.522^NS^
Lymphocyte (%)	82.75 ± 2.50	76.75 ± 2.93	79.25 ± 1.11	75.25 ± 3.35	77.00 ± 3.11	0.370^NS^
Monocyte (%)	1.00 ± 0.41	1.50 ± 0.96	0.75 ± 0.25	0.75 ± 0.48	2.50 ± 0.41	0.461^NS^
Eosinophil (%)	0.25 ± 0.25	0.50 ± 0.29	0.50 ± 0.50	0.50 ± 0.29	0.50 ± 0.29	0.977^NS^
Basophil (%)	0.00 ± 0.00	0.50 ± 0.29	0.00 ± 0.00	0.50 ± 0.29	1.00 ± 0.58	0.192^NS^
H:L	0.20 ± 0.03	0.28 ± 0.06	0.25 ± 0.02	0.32 ± 0.07	0.26 ± 0.04	0.498^NS^

^a,b^Row means with distinct alphabets differ significantly at *P* < 0.05 (One-Way ANOVA, Duncan's multiple range test); PCV: Packed cell volume, HB: hemoglobin, RBC: red blood cell, WBC: white blood cell; H:L: Heterophil: Lymphocyte ratio; NS: Not significant; DEX: Dexamethasone; VE: Vitamin E; Se: Selenium.

### Blood glucose and cholesterol

The effects of VE and Se supplementation on blood glucose and cholesterol levels (± SEM) in DEX-stressed quail cocks are presented in Table 4. As observed, there were no significant differences (*P* > 0.05) in blood glucose or cholesterol concentrations among the treatment groups.

**Table 4:** Effect of dietary vitamin E and selenium on blood glucose and cholesterol of dexamethasone (± SEM) stressed quail cock.

Parameter	Group 1 (Control)	Group 2 (DEX)	Group 3 (DEX + VE)	Group 4 (DEX + Se)	Group 5 (DEX + VE + Se)	P-value
Glucose (mg/dl)	77.0 ± 3.11	84.0 ± 2.86	77.75 ± 4.09	78.00 ± 5.82	73.50 ± 6.29	0.627^NS^
Cholesterol (mg/dl)	79.75 ± 2.10	82.25 ± 1.31	87.00 ± 2.12	84.00 ± 2.04	88.50 ± 0.65	0.053^NS^

NS: Not significant (*P* ≥ 0.05; One-Way ANOVA, Duncan's multiple range test); DEX: Dexamthasone; VE: Vitamine E; Se: Selenium.

### Plasma antioxidant status

**Fig. 2:**
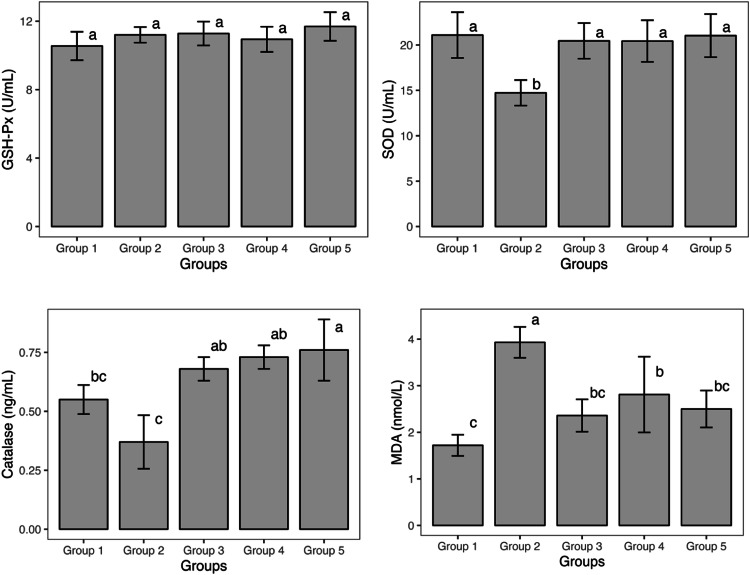
Effect of vitamin E and selenium on plasma antioxidant status of dexamethasone stressed quail ^a-c^Means each chart with distinct alphabets differ significantly (*P* < 0.05; One-Way ANOVA, Duncan's multiple range test); GSH-Px: glutathione peroxidase; SOD: Superoxide dismutase, MDA: malondialdehyde; Group 1: negative control, Group 2: DEX-treated (0.1 mg/per bird), Group 3: DEX + VE (180 mg diet^−1^); Group 4: DEX + Se (0.3 mg diet^−1^); and Group 5: DEX + VE (180 mg diet^−1^) + Se (0.3 mg diet^−1^).

Analysis of plasma antioxidant status in quail cocks revealed significant variations (*P* < 0.05) in the plasma levels of SOD and CAT, as well as MDA, a marker of lipid peroxidation (Fig. 2). GSH-Px activity, however, remained unaffected (*P* ≥ 0.05). Cocks in the control group (G1) displayed the highest SOD activity, which was similar (*P* ≥ 0.05) to those in the G3, G4, and G5 groups. Conversely, the DEX-treated group (G2) exhibited the lowest SOD activity, significantly different from all other groups. Similar trends were observed for CAT activity. G2 had the lowest CAT activity, although not statistically different from G1. G3 and G4 displayed CAT activity comparable to the control, but significantly higher than G2. Interestingly, the VE+Se group (G5) had the highest CAT activity, exceeding G1 and G2 but statistically similar to G3 and G4. Notably, complete reversal to control levels was observed for SOD activity in the VE (G3), Se (G4), and combined VE+Se (G5) groups. Interestingly, the combined supplementation showed the most significant improvement in CAT activity.

MDA levels were highest (*P* < 0.05) in the DEX-treated group, indicating increased lipid peroxidation compared to all other groups. Conversely, the control cocks showed the lowest MDA levels, statistically similar to the G3 and G5 groups. Overall, MDA values significantly improved following VE, Se, and VE+Se treatment compared to the DEX group, with G3 (VE) and G5 (VE+Se) showing near-complete recovery.

### Seminal characteristics and oxidative stress status

Table 5 presents the semen quality characteristics of DEX-stressed quail cocks. Either DEX stress or supplementations with VE and Se did not significantly affect (*P* ≥ 0.05) semen volume, sperm concentration, viability, percentage of dead sperm, and seminal OS markers of semen glutathione peroxidases and semen MDA. However, spermatozoa motility was affected (*P* < 0.05) across the treatment groups. G1, G3, G4, and G5 exhibited significantly higher and similar motility compared to DEX-treated (G2). Cocks in the negative control group (G1) displayed statistically similar (*P* ≥ 0.05) motility to cocks in DEX + VE group (G3), DEX + Se group (G4), and the DEX + VE + Se group (G5). These groups exhibited higher sperm motility than the DEX-only-treated group (G2).

**Table 5:** Effect of vitamin E and selenium supplementation on semen quality characteristics and oxidative stress markers (± SEM) of dexamethasone stressed quail cocks.

Indices	Group 1 (Control)	Group 2 (DEX)	Group 3 (DEX + VE)	Group 4 (DEX + Se)	Group 5 (DEX + VE + Se)	SEM	P-value
Volume (uL)	22.10 ± 1.08	18.13 ± 0.75	22.37 ± 0.64	21.67 ± 1.65	21.80 ± 1.10	0.59	0.109^NS^
Conc. (× 10^6^/mL)	502.88 ± 37.51	298.32 ± 51.36	426.05 ± 81.83	404.31 ± 75.38	456.31 ± 99.65	28.07	0.202^NS^
Motility (%)	73.33^a^ ± 1.67	60.00^b^ ± 5.0	75.00^a^ ± 5.0	70.00^a^ ± 0.0	75.00^a^ ± 2.89	1.88	0.025*
Viability (%)	78.33 ± 4.41	68.33 ± 3.33	71.67 ± 9.28	78.33 ± 4.41	76.67 ± 6.01	2.46	0.684^NS^
Dead Sperm (%)	21.67 ± 4.41	31.67 ± 3.33	28.33 ± 9.28	21.67 ± 4.41	23.33 ± 6.01	2.46	0.684^NS^
sGSH-Px (U/mL)	2.39 ± 0.59	3.84 ± 0.19	3.74 ± 0.35	3.79 ± 0.47	3.97 ± 0.60	0.23	0.153^NS^
sMDA (nmol/L)	9.49 ± 0.61	9.68 ± 0.44	10.04 ± 0.63	10.16 ± 0.64	10.63 ± 0.55	0.25	0.674^NS^

^a,b^Row means with distinct alphabets differ significantly at *P* < 0.05 (One-Way ANOVA); Conc.: Spermatozoa concentrations; sGSH-Px: Semen glutathione peroxidases; sMDA: semen malondialdehyde; NS: Not significant; DEX: Dexamethasone; VE: Vitamin E; Se: Selenium.

## Discussion

Our findings revealed that DEX supplementation (G2) suppressed WG, TFI, and DFI relative to the control (G1). This decrease in WG and DFI aligns with previous studies in Japanese quails (Berenjian et al., 2018) and broiler chickens (Ademu et al., 2018; Afrose et al., 2018) exposed to DEX and may be attributed to increased protein catabolism, muscular dystrophy, and mobilization of fat stores. Physiological stress (such as heat or corticosterone treatment) can stimulate the release of corticosteroids in birds, negatively impacting their growth process (Bohler et al., 2021). Song et al. (2011) reported that DEX elevates circulating urate, a marker of enhanced protein catabolism, leading to reduced muscle growth and body mass. Birds in G3 (DEX + VE), G4 (DEX + SE), and G5 (DEX + VE + SE) exhibited similar WG with the control (G1) and higher WG compared to the DEX-only group (G2). Notably, a reversal in the DEX-induced decline in TFI and DFI was observed in the VE- and Se-supplemented groups (G3, G4, and G5). The significant improvements in WG observed in G3, G4, and G5 were associated with improved TFI and DFI, suggesting that VE and Se can mitigate the negative impacts of DEX by restoring appetite and, consequently, enhancing the performance of stressed Japanese quails. Dietary supplementations with antioxidants including vitamins E and C have been reported to boost overall body metabolism by protecting against oxidants- and inflammatory cytokines-induced cellular damage (Jang et al., 2014; Min et al., 2018). These results are in tandem with those of Calik et al. (2022), who reported that VE and Se supplementation in broilers improved body WG and FI under stressful environmental conditions. Other studies have also shown dietary chromium supplementation ameliorated the DEX-induced decreased FI and body WG in Japanese quails (Onderci et al., 2005; Berenjian et al., 2018). Similarly, Sahin and Kucuk (2001) combined VE (250 mg kg^−1^) and Se (0.2 mg kg^−1^) supplementation enhanced quail performance under sustained HS, suggesting a potential synergy between these two micronutrients in alleviating OS. Positive synergistic effects and improvements in FI and growth were also reported when VE was combined with vitamin C in Japanese quails (Khazaei et al., 2021). Contrastingly, earlier studies have failed to demonstrate the efficacy of VE and/or Se to enhance growth during stress. Lower IBW and FI were observed in Japanese quails fed nano-Se supplementation at 0.2 mg/kg diet (El-Kazaz et al., 2020). Habibian et al. (2016) observed no marked variations in performance between control birds and those supplemented with VE (0, 125, or 250 mg kg^−1^) and Se (0, 0.5, or 1 mg kg^−1^) under stress. These variations may be due to the form of antioxidant used, the species of bird, or the form of stressor applied. Overall, the findings of this study revealed that while DEX administration significantly suppressed FI and WG in breeder quail cocks, dietary supplementation with VE, Se, or their combination appeared to mitigate these adverse effects, potentially by counteracting the appetite-suppressing and catabolic effects of DEX.

The haematological profile of birds offers valuable insights into their physiological state and serves as a sensitive marker for responses to internal or external stressors (Ekunseitan et al., 2021). In the present study, DEX administration increased (*P* < 0.05) Hb and PCV compared to the control group (G1). Hb, the oxygen-transporting molecules in RBC, directly reflects the blood's oxygen-carrying capacity. PCV, the fraction of blood occupied by RBC, is another Hb indicator (Zachariah et al., 2018). In this study, the administration of dexamethasone resulted in significant increases in Hb and PCV levels in dexamethasone-stressed quail birds. This is an intriguing observation since stress typically correlates with increased OS, which results in increased hemolysis and decreased erythropoiesis, leading to a reduction in these parameters (Zulkifli et al., 2009; Wang et al., 2018; Goodchild et al., 2022). Our observed increase in Hb and PCV of DEX-treated birds in this study may be attributed to stress erythropoiesis, a well-documented adaptative phenomenon involving increased RBC production in response to stressors like hypoxia and anemia. DEX, an immunosuppressant, may induce stress, elevating metabolic demands. The birds may compensate by enhancing oxygen-carrying capacity through increased Hb, improving their ability to meet heightened energy needs and aid in mitigating the detrimental impacts of stress-induced oxidative damage. This adaptative response is crucial for maintaining metabolic functions under stress and highlights avian resilience and physiological flexibility (Gorelik et al., 2020; Nwaigwe et al., 2020). Supporting this concept, Goodchild et al. (2022) reported higher Hb concentrations in rural song sparrows with lower body condition scores, suggesting greater metabolic demands as an adaptation to environmental stressors. Previous studies have also indicated that birds can modify Hb concentrations to meet heightened energy requirements (Jaeger and McGrath, 1974; Prats et al., 1996; Bury et al., 2019). Also, the increased Hb and PCV may suggest hemoconcentration conditions arising from possible DEX-induced dehydration. One of the primary roles of corticosteroids in stress responses is the regulation of electrolyte and water balance. Corticosteroids achieve this by reducing water intake and promoting diuresis, which limits the expansion of extracellular fluid compartments (Liu et al., 2010; 2016). Gorelik et al. (2020) demonstrated that stress exposure in chickens stimulated erythropoiesis and elevated blood Hb levels. Similarly, Aengwanich (2007) reported significant increases in Hb and PCV in broiler chickens subjected to DEX administration for 21 days, which aligns with our findings. Other studies (Nwaigwe et al., 2020; Ademu et al., 2024) have also observed increased Hb concentrations in chickens exposed to feed withdrawal and DEX-induced stress, respectively. Contrastingly, studies have reported reduced PCV and Hb levels following DEX administration. Yarmohammadi et al. (2020) noted such declines in Japanese quail, while Mushi et al. (1999) documented similar effects in pigeons. These discrepancies highlight the variability in hematological responses to stress in birds, potentially influenced by species, duration of exposure, and experimental conditions.

WBC counts markedly increased in all DEX-treated groups as compared to the control. This is attributable to the anti-inflammatory and immunomodulatory attributes of DEX, known to elevate WBC count (glucocorticoid-induced leukocytosis) (Frenkel et al., 2019). Heterophils and lymphocytes are the primary circulating avian leukocytes. The heterophil-to-lymphocyte **H:L**) (ratio is a recognized stress/immunosuppression marker (Davis et al., 2008; Hõrak et al., 2013; Nazar et al., 2018). The WBC increase is primarily due to a rise in circulating heterophils through both genomic and non-genomic mechanisms, including stimulating bone marrow production while modulating other leukocyte populations (Cheng, 2018; Jia and Zhang, 2022). Earlier reports have established a strong correlation between glucocorticoid administration, heterophilia, and lymphopenia in birds, leading to an increased H:L ratio (McFarlane and Curtis, 1989). While we observed trends toward increased heterophils and H:L ratio with decreased lymphocytes following DEX administration, these variations were not statistically significant (*P* > 0.05). These non-significant differences may be due to factors such as acclimation or a waning of DEX-induced leukocyte changes, as reported in House Finches (*Haemorhous mexicanus*) (Crouch et al., 2022). Aengwanich and Chinrasri (2003) reported that in Japanese quails, lower doses of DEX (1.25 to 2.5 mg/kg) administration increased heterophils, decreased lymphocytes, and elevated heterophil-to-lymphocyte (H:L) ratios. However, higher DEX doses (5 mg/kg) did not significantly affect these indices. This further supports a possible acclimation and the involvement of complex physiological mechanisms in avian responses to DEX, requiring further elucidation. Our findings align with previous reports of elevated Hb, PCV, and WBC in DEX-treated broiler chickens (Pyrrho et al., 2004; Aengwanich, 2007). However, in contrast to our findings, Yarmohammadi et al. (2020) and Berenjian et al. (2018) reported decreased levels of Hb, PCV, and WBC, whereas Vicuña et al. (2015) noted no changes in WBC among Japanese quails administered DEX. Lowered blood PCV, Hb (Wang et al., 2018), and WBC (Mahmoud et al., 2013) have also been previously reported in heat-stressed Japanese quails and broiler chickens. These discrepancies highlight the potential influence of environmental variations, bird strain/species, and experiment duration on the hematological response to DEX.

Interestingly, dietary supplementation with VE, Se, or their combination (VE + Se) did not modify the observed DEX-induced changes in Hb and WBC of the quail cocks. Se supplementation (G4) or its combination with VE (G5) seemed to exacerbate the PCV changes compared to the control. The exact reasons for this are unclear, but while antioxidants like VE and Se effectively mitigate OS, they may not directly influence other stress-affected pathways, such as corticosterone-driven energy partitioning, immune suppression, or hematological changes. Additionally, quails’ relative resilience to stress may reduce the observable benefits of antioxidant supplementation compared to more stress-sensitive species. Our findings align with previous studies showing that while physiological stress adversely affects performance and hematological indices in Japanese quails, dietary supplementation with nanoparticle-chromium, chromium-methionine, and zinc-proteinate had no significant impact on stressed birds (Yarmohammadi et al., 2020). Nevertheless, our findings are in contrast with previous studies reporting the influence of VE and Se on avian hematology. Ekunseitan et al. (2021) observed decreases in PCV, Hb, and RBC counts in broiler chickens fed dietary VE and Se, suggesting a direct effect on blood cell production. Conversely, Tayeb and Qader (2012) reported that moderate VE and Se supplementation levels improved HB concentrations, potentially enhancing the erythropoietic stress response (Ekunseitan et al., 2021). Additionally, Berenjian et al. (2018) demonstrated a reversal of DEX-induced changes in Hb, PCV, and WBC in Japanese quails with increasing dietary chromium levels. These contrasting results lend further credence to the presence of a complex response mechanism of quail cocks under chronic physiological stress, and dietary antioxidants.

In this study also, we observed that neither DEX administrations nor supplementation with VE and Se had a significant impact on the quail birds' blood glucose and cholesterol levels. These findings are noteworthy as our findings are contrary to the previously established link between glucocorticoid/DEX administration and elevated blood glucose and cholesterol in birds (Detka et al., 2015; Buning et al., 2017; Lv et al., 2018; Hosny et al., 2020). While the reason for this remains unclear, DEX is acknowledged for its role in modulating stress response and metabolism regulations. However, its effects on avian physiology and metabolism can vary depending on the species, as well as the administration route and dosages. Regarding quails, while studies have indicated that DEX can alter growth and various metabolic pathways, higher administration doses were not observed to cause any changes in several physiological indicators (Aengwanich and Chinrasri, 2003). This suggests a possible threshold effect where only lower administration doses could elicit certain observable changes in the blood biochemistry. Additionally, as a tropical avian species (Barbosa Lima et al., 2014), quails may have evolved unique regulatory mechanisms for maintaining glucose and lipid metabolism that are less susceptible to the fluctuations of stressful conditions such as DEX exposure or even the tested supplements. Earlier reports have recognized the relatively higher tolerance of quails to stress compared to other avian species, such as chickens (Barbosa Lima et al., 2014). They may, therefore, inherently possess higher basal metabolic rates and more efficient glucogenic pathways, which could buffer against the hyperglycemic or hypercholesteremic effects often associated with glucocorticoids. Our findings align with earlier reports indicating that chronic DEX administration did not affect plasma glucose in young quails (Bray, 1993). On the other hand, contrasting reports have documented elevated glucose and/or cholesterol levels in Japanese quails or chickens following oral DEX treatment, dietary DEX supplementation, or HS (Kobayashi et al., 1989; Abou El-Soud et al., 2006; Lv et al., 2018; Sultana et al., 2021).

On the other hand, VE and Se are both known for their antioxidant properties and their roles in various metabolic processes. However, their supplementations in the present study did not influence blood glucose or cholesterol in the DEX-treated quails. Studies have shown that while these nutrients can improve general health and immune functions, their direct impacts on metabolic indices can be limited. For instance, dietary Se supplementation did not cause any significant changes in the blood glucose and cholesterol of naked-neck chickens, despite their impact on other performance indices (Khan et al., 2022). This observation may be attributed to the presence of a robust physiological capacity to modulate and regulate these metabolic indices in tropical birds, regardless of dietary supplementation. Furthermore, the regulation of blood glucose and cholesterol levels is under the tight control of the endocrine system, which involves insulin and glucagon, as well as the liver. If a healthy animal has these hormonal pathways functioning optimally, the impact of external factors such as DEX or vitamin and mineral supplements may not result in any observable changes in these systems. it is important to note that the quails used for this study were healthy and well-fed, which may have contributed to the maintenance of homeostasis in some key metabolic parameters. Our findings are, however, in contrast with Gursu et al. (2002), who reported lower blood glucose and cholesterol in HS quails supplemented with these vitamins. Additionally, Abd El-Hack et al. (2017) observed increased cholesterol with Se and vitamin A supplementation in laying hens, while Habibian et al. (2014) found that VE and Se lowered blood glucose and cholesterol only in heat-stressed chickens, not under thermoneutral conditions. On the other hand, El-Sebai (2000) reported increased cholesterol with dietary VE and Se supplementation in broiler chickens. The discrepancies between these studies and the results of the present findings suggest the involvement of various factors such as the age and genetics of the birds, environmental conditions, feeding management, dosage, duration, and means of DEX administration, as well as the form and dosage of VE and Se used, in avian responses to stress and antioxidants.

Depending on the exposure duration, DEX induces OS in Japanese quails and chickens (Hanafy and Khalil, 2015; El-Senousey et al., 2018). Birds possess a robust antioxidant defense mechanism involving both enzymatic and non-enzymatic components to counteract OS (Cheng et al., 2017). Key antioxidant enzymes include SOD, CAT, and GSH-Px. SOD, primarily present in the cytosol (Cu/Zn-SOD) and mitochondria (Mn-SOD) acts as the initial line of defense, dismutating superoxide radicals into hydrogen peroxide (H_2_O_2_) and oxygen (O_2_) (Yamaguchi et al., 1994). CAT, on the other hand, plays a key role in the detoxifying H_2_O_2_ into water and O_2_ (Turgay et al., 2012). Similarly, GSH-Px, a Se-containing metalloenzyme widely distributed in the cytoplasm and mitochondria, is involved in detoxifying H_2_O_2_ into water and oxygen (Cichoski et al., 2012; Rahal et al., 2014). MDA is a consistent measure of lipid peroxidation, a consequence of OS (Sahin et al., 2003). These blood indices have long been used as reliable indicators of OS status in birds. In the present study, we observed a significant reduction in the levels of SOD and CAT, alongside an increase in MDA levels following DEX administration to the quails, indicating an increase in oxidative stress. These results align with studies showing DEX-induces decrease in SOD activity and elevation in MDA levels, indicative of increased OS (Hanafy and Khalil, 2015; Lv et al., 2018; El-Senousey et al., 2018; Fathi et al., 2023). DEX exerts its influence on OS through the hypothalamic-pituitary-adrenal axis, altering metabolism (reducing antioxidant enzyme activity) and promoting free radical production (Craig and Craig, 1985; Lin et al., 2004). Supporting this, studies in mice have shown that prolonged DEX exposure increases glucocorticoid secretion while diminishing antioxidant enzyme activity in the liver and skeletal muscle, ultimately leading to lipid peroxidation and worsening liver steatosis, especially under a fatty diet (Pereira et al., 1999; Poggioli et al., 2013; Lv et al., 2019). Interestingly, in contrast to the observed changes in the levels of SOD, CAT, and MDA, the administration of DEX did not appear to alter blood GSH-Px activities in the present study. Consistent with these findings, previous research has shown that while DEX significantly affected the levels of various antioxidant enzymes (*P* < 0.05), GSH-Px activities remained unchanged across different tissues, including the gastric mucosa and myocardium (Manjari and Das, 2000; Devi et al., 2007). This suggests that GSH-Px may be less sensitive to DEX-induced oxidative stress compared to SOD and CAT. Moreover, unlike SOD and CAT, which are directly involved in neutralizing reactive ROS, GSH-Px utilizes reduced glutathione (GSH) as a substrate. Consequently, its activity is largely dependent on the availability of GSH and its selenium cofactor. In the context of this study, the baseline levels of GSH and selenium in the quails may have been sufficient to sustain GSH-Px activity, making it less susceptible to fluctuations induced by DEX or antioxidants.

In this study, dietary supplementation with VE and Se, well-known antioxidants, aimed to mitigate DEX-induced OS by enhancing antioxidant defense mechanisms in quail cocks. While GSH-Px activity remained unchanged, both SOD and CAT activities exhibited a positive response. Supplementation with VE, Se, or their combinations appeared to ameliorate or reverse the DEX-induced changes in SOD, CAT, and MDA levels. These findings demonstrate that VE and Se supplementation in quails' diets can alleviate the negative impacts of DEX-induced OS on plasma antioxidant enzymes and the OS marker, MDA. DEX administration mimics the stress-induced increase in catecholamine and corticosteroid secretion in birds, leading to decreased antioxidant enzyme activity, elevated OS (Lin et al., 2004), and increased lipid peroxidation (Gao et al., 2010; Yarmohammadi et al., 2020). VE and Se likely exert their protective effects by stimulating and supporting antioxidant enzymes’ activity, thereby bolstering cellular defense mechanisms against ROS production and subsequent membrane deterioration (El-Senousey et al., 2018; Shakeri et al., 2020; Calik et al., 2022). VE and Se act as a powerful duo in combating OS in birds. VE functions as a cellular enzymatic regulator and a lipid-soluble antioxidant. It safeguards cell membrane integrity by minimizing the oxidation of polyunsaturated fatty acids, a process that can generate harmful free radicals (Traber and Stevens, 2011; Nimse and Pal, 2015). Similarly, Se plays a vital role by being incorporated into antioxidant enzymes like GSH-Px. These enzymes directly scavenge free radicals and reduce the formation of byproducts like lipoperoxidation products (Sahin and Kucuk, 2001; Habibian et al., 2015). Interestingly, Se also helps preserve the functions of key antioxidant enzymes, particularly SOD and CAT (Tsekhmistrenko et al., 2020). This complementary action of VE and Se creates a robust defense system against OS, ultimately promoting cellular health. Previous research has shown the effectiveness of VE and Se in mitigating various forms of OS in avian species (Li et al., 2014; Yang et al., 2014; Cheng et al., 2017). Our findings concur with previous reports, showing that VE supplementation and Se, either alone or combined with other antioxidants, can improve the adverse impacts of DEX or HS on antioxidant enzyme activity and OS markers in Japanese quails and chickens (Eid et al., 2006; Gao et al., 2010; El-Senousey et al., 2018). Additionally, VE has been shown to alleviate the detrimental effects of dietary copper-induced OS in chickens (Cinar et al., 2014). In this study, we report for the first time that combined dietary supplementation with VE and Se significantly enhances antioxidant enzyme activities, thereby mitigating chronic DEX- or stress-induced OS in quail birds.

Sperm assessment has become an indispensable tool in the poultry industry, providing a reliable method for monitoring male fertility and overall reproductive performance (Sarica et al., 2007). Stressors, including high-temperature conditions, can adversely affect sperm quality and reproductive outcomes through elevated glucocorticoid levels (Edens and Siegel, 1975; Kalamah, 2001; Eid et al., 2006). In our study, we observed a significant (*P* < 0.05) decline in sperm motility in the DEX-treated group (G2), accompanied by a marginal reduction in sperm concentration compared to the control group. Notably, there were no significant alterations in other semen quality indices, including volume, sperm concentration, viability, or the seminal plasma OS markers, sGSH-Px and sMDA. This finding is particularly intriguing given previous reports linking increased oxidative stress and insufficient seminal antioxidant activity to declining semen quality (Shiva et al., 2011). DEX is known to alter the antioxidant composition of seminal plasma, potentially disrupting the microenvironment necessary for optimal sperm function (Sadeghzadeh et al., 2018). However, sperm motility is influenced by various factors beyond antioxidant status, including energy metabolism, the structural integrity of sperm cells, and the overall microenvironment within the reproductive tract (Barbagallo et al., 2020). In this study, DEX may have directly affected sperm cells or triggered metabolic changes related to hormonal balance, seminal pH, ion concentration, or seminal viscosity. These alterations could negatively impact sperm motility independently of oxidative stress or antioxidant activity in seminal plasma. Specifically, DEX administration can mimic the adverse effects of elevated blood glucocorticoid levels by inhibiting the release of Gonadotropin-Releasing Hormone (GnRH) and pituitary gonadotropins such as luteinising hormone (LH) and follicle-stimulating hormone (FSH) (Sapolsky et al., 2000; Eid et al., 2006; Hatamoto et al., 2006). This inhibition may decrease Leydig cell function and subsequently reduce androgen production in breeder quails (Hanafy and Khalil, 2015; Semet et al., 2017). Lower testosterone levels can impair sperm motility by altering seminal plasma composition, disrupting mitochondrial activity and ATP production essential for flagellar movement, and downregulating proteins critical for flagellar function and overall motility (Yamanaka et al., 2024). Furthermore, DEX treatment may induce OS by stimulating reactive oxygen species ROS generation while concurrently reducing the activities of endogenous antioxidant enzymes such as CAT, SOD, and GSH-Px. The excessive ROS produced could lead to increased lipid peroxidation in various organs of the reproductive system, including the testes and sperm membranes. This may compromise their integrity and functionality, ultimately diminishing overall sperm production and quality (Eid et al., 2006). Additionally, DEX has been shown to cause atrophy of seminiferous tubules, disrupting spermatogenesis and increasing the production of deformed sperm cells with compromised motility (Hanafy and Hassan, 2021). These findings are consistent with previous studies demonstrating that DEX treatment reduces serum testosterone levels, adversely impacting spermatogenesis and sperm motility in mice (Sadeghzadeh et al., 2019). Our results also align with earlier reports indicating that chickens exposed to DEX-induced stress experienced detrimental effects on sperm concentration and motility (Eid et al., 2006). However, contrary to the findings of Eid et al. (2006), our study did not reveal a significant impact on the percentage of dead sperm cells.

Chronic stress can adversely affect avian reproduction, but the permanence of these effects remains uncertain (Hanafy and Khalil, 2015). Our study indicates that supplementation with VE and Se may mitigate the detrimental effects of DEX on sperm motility. Quail cocks receiving VE, Se, or a combination of both exhibited significantly improved sperm motility compared to the DEX-only group. These enhancements in motility, despite no changes in other semen quality indices, may result from improved hormone and energy production or metabolic efficiency rather than a direct increase in seminal antioxidant activity. Both VE and Se are potent antioxidants that modulate endocrine secretion and reproductive functions in birds (Yoon et al., 2007; Moslemi and Tavanbakhsh, 2011; Ebeid, 2012; El-Kazaz et al., 2020). Se is a critical component of GSH-Px and other selenoproteins essential for spermatogenesis, sperm function, and testosterone biosynthesis (Olson et al., 2005; Marin-Guzman et al., 2000; Khalaf et al., 2019; Lovas et al., 2010). Research has shown that Se protects testicular tissues from oxidative damage while enhancing plasma testosterone levels and semen quality in Japanese quails and chickens (Cai et al., 2012; El-Kazaz et al., 2020). Deficiencies in Se can lead to sperm abnormalities, including reduced motility and abnormal morphology (Wu et al., 2003). VE also plays a vital role as an antioxidant by mitigating the harmful effects of free radicals on reproductive tissues, promoting their growth and development (Traber, 1999; El-Azzazi et al., 2016). Additionally, VE may influence the density of spermatogenic cells within the testes, further enhancing sperm production (Cheah and Yang, 2011). VE often works synergistically with Se to amplify their beneficial effects on metabolism and reproductive health (Awadeh et al., 1998; Sahin et al., 2002). Our findings corroborate previous research demonstrating VE's positive impact on semen quality in Japanese quails (Adabi et al., 2011). Eid et al. (2006) also reported a dose-dependent improvement in semen quality among DEX-stressed chickens supplemented with VE. Moreover, dietary Se supplementation has been shown to counteract the negative effects of DEX (Khalil-Khalili et al., 2021) and heat stress (Ebeid, 2009) on chicken semen quality. VE and Se have been reported to synergistically enhance semen production in rabbit bucks (El-Azzazi et al., 2016) while alleviating high-temperature stress effects on chicken semen (Ebeid, 2012). Overall, our study demonstrates that supplemental VE and Se can mitigate the negative impacts of DEX on sperm motility, suggesting their potential as protective agents against stress-induced reproductive impairments in birds. Interestingly, we observed no significant changes in seminal oxidative stress markers following DEX or antioxidant treatments. Although the reason for this discrepancy is unclear, we hypothesize that the mechanisms by which these supplements exert their effects may also function independently of seminal antioxidant parameters. This aligns with findings by Alahmar et al. (2023), who reported no significant effects of DEX (0.2 mg/kg/day) or coenzyme Q10 on testicular antioxidant capacity markers such as CAT and SOD in rats. Prior studies indicate that supplementation with VE, Se, or their combination can enhance antioxidant enzyme activity such as GSH-Px and reduce MDA levels in the seminal plasma of birds under physiological stress (Eid et al., 2006; Khalil-Khalil et al., 2023). The inconsistencies between our findings and previous reports may stem from differences in DEX dosage or administration methods, species studied, environmental conditions, or management practices. Our findings further support our hypothesis regarding the complex interplay of mechanisms that influence the effects of stress or glucocorticoids, as well as the impact of supplemental vitamin E (VE) and selenium (Se), on the overall health and productivity of Japanese quail. This underscores the need for further research to elucidate these interactions.

## Conclusion

Our findings reveal that dietary supplementation with VE and Se effectively mitigated DEX-induced oxidative stress and reproductive impairment in Japanese quail cocks. Including these antioxidants in the diet can offer a practical strategy for enhancing the welfare and productivity of quails under stressful conditions.

## Declaration of generative artificial intelligence (AI) and AI-assisted technologies in the writing process

The author(s) utilized artificial intelligence tools like ChatGPT and Google Gemini to enhance the manuscript's readability and correct grammar during its preparation. After employing this tool/service, the author(s) critically reviewed and amended the content as necessary, assuming complete responsibility for the publication's accuracy and integrity.

## Disclosures

The authors declare that they have no known competing financial interests or personal relationships that could have appeared to influence the work reported in this paper.
